# Circulating Organ-Specific MicroRNAs Serve as Biomarkers in Organ-Specific Diseases: Implications for Organ Allo- and Xeno-Transplantation

**DOI:** 10.3390/ijms17081232

**Published:** 2016-08-01

**Authors:** Ming Zhou, Hidetaka Hara, Yifan Dai, Lisha Mou, David K. C. Cooper, Changyou Wu, Zhiming Cai

**Affiliations:** 1 Shenzhen Xenotransplantation Medical Engineering Research and Development Center, Shenzhen Second People’s Hospital, First Affiliated Hospital of Shenzhen University, Shenzhen 518039, China; zhouming2004@126.com; 2 Institute of Immunology, Zhongshan School of Medicine, Guangdong Provincial Key Laboratory of Organ Donation and Transplant Immunology, Sun Yat-sen University, Guangzhou 510275, China; changyou_wu@yahoo.com; 3 Thomas E. Starzl Transplantation Institute, University of Pittsburgh, Pittsburgh, PA 15261, USA; harah@upmc.edu (H.H.); coopdk@upmc.edu (D.K.C.C.); 4 Jiangsu Key Laboratory of Xenotransplantation, Nanjing Medical University, Nanjing 210029, China; daiyifan@njmu.edu.cn

**Keywords:** allotransplantation, biomarker, immune rejection, miRNA, circulating, xenotransplantation

## Abstract

Different cell types possess different miRNA expression profiles, and cell/tissue/organ-specific miRNAs (or profiles) indicate different diseases. Circulating miRNA is either actively secreted by living cells or passively released during cell death. Circulating cell/tissue/organ-specific miRNA may serve as a non-invasive biomarker for allo- or xeno-transplantation to monitor organ survival and immune rejection. In this review, we summarize the proof of concept that circulating organ-specific miRNAs serve as non-invasive biomarkers for a wide spectrum of clinical organ-specific manifestations such as liver-related disease, heart-related disease, kidney-related disease, and lung-related disease. Furthermore, we summarize how circulating organ-specific miRNAs may have advantages over conventional methods for monitoring immune rejection in organ transplantation. Finally, we discuss the implications and challenges of applying miRNA to monitor organ survival and immune rejection in allo- or xeno-transplantation.

## 1. Introduction

### 1.1. Current Status of Biomarkers in the Detection of Graft Rejection

Organ failure is the leading cause of human death [1]. Replacing a non-functioning organ with a new one is the most effective strategy to cure terminal organ failure and maintain patients’ lives. However, immune rejection still remains a major obstacle for long-term survival of allo- or xeno-transplanted organs (reviewed in [2]). Real-time monitoring of immune rejection is critical for organ allo (xeno)-transplantation. However, existing methods are limited. Currently, detection of acute rejection is largely based on clinical data such as a patient’s symptoms and physical signs, but also on laboratory data such as biochemical and immunological assays and tissue biopsies [3], some of which are not ideal for clinical application. A patient’s symptoms and physical signs are often of value only at a ‘late-stage’ of rejection. Tissue biopsies are the ‘gold standard’, but are invasive and expensive with some limitation of diagnostic accuracy [3,4]. Biochemical and immunological assays are either of low sensitivity or low specificity [5,6].

For example, biochemical biomarkers of serum creatinine and urine albumin are classical indicators of kidney injury following renal allo (xeno)-transplantation, but are of relatively low sensitivity (reviewed in [7]). Furthermore, there are no reliable methods or biomarkers to monitor chronic rejection or acute-on-chronic rejection. Apart from immune rejection, there are special concerns in organ allo (xeno)-transplantation, such as recurrence of primary disease, thrombosis formation, and hemolysis, all of which need to be properly monitored post-transplantation. Therefore, novel non-invasive biomarkers that might specifically monitor immune rejection and/or rejection-associated complications are urgently needed. Circulating miRNAs may serve as non-invasive, specific, sensitive, and low-cost biomarkers in organ allo (xeno)-transplantation.

### 1.2. Potential of Circulating miRNAs as Biomarkers of Graft Rejection

MicroRNA (miRNA) is a small non-coding RNA containing approximately 22 nucleotides; it is found in different species and functions in the RNA silencing and post-transcriptional regulation of 30% of the gene expression in humans [8,9], including proliferation, DNA repair, differentiation, metabolism, and apoptosis [10,11]. Different cells/tissues/organs possess different miRNA expression profiles [12,13]. Some miRNAs are specific or abundant in certain organs, e.g., miR-122 is liver-specific [13]. MiRNA expression profiles, including cell/tissue/organ-specific miRNAs, indicate different diseases, such as cancers [14]. Profiles of miRNA in the body fluids, also called circulating miRNA or cell-free miRNA, serve as non-invasive biomarkers for physiological and pathological changes, such as cancers [15], organ injury [16], diabetes [15], and even pregnancy [17,18].

**Figure 1:**
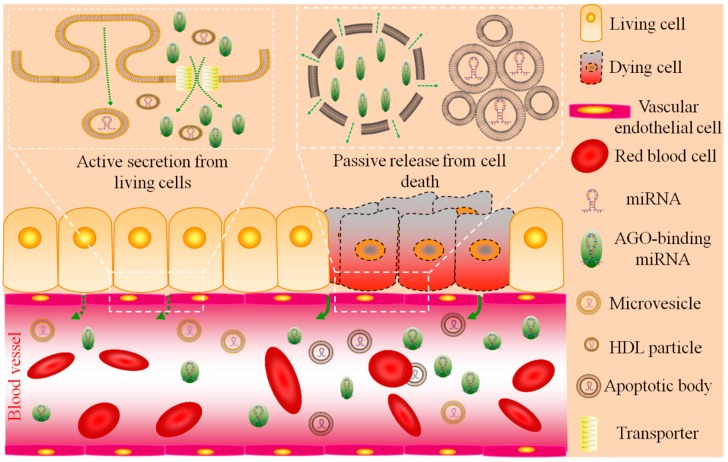
A schematic model of sources of circulating miRNAs. Circulating miRNAs can be actively secreted from living cells, mainly in the form of microvesicles and AGO-binding miRNA derived from the exosome pathway and transmembrane transporter, respectively. They can also be passively released from dying cells in the form of necrosis lysate or apoptotic bodies. All the cell-free miRNAs finally diffuse into body fluids, such as the blood. Solid and broken green arrows between vascular endothelial cells indicate large-scale and micro-scale release of circulating miRNA, respectively. All the source materials were obtained from a web-accessible software plugin of PowerPoint: Science Slide 5.

Cell death, such as apoptosis or necrosis, is the final result of immune rejection. Theoretically, intracellular miRNAs are passively released from rejected cells and become part of the circulating miRNAs (reviewed in [19]) (also shown in Figure 1). In fact, this is the scientific basis for most of the rejection-related biomarkers, including proteins, DNA, and RNA, which follow cell death once the allo (xeno)-grafts are attacked by the host immune system. Both proteins and DNA are proved to be useful biomarkers for acute immune rejection [4,20,21,22,23,24]. As described above, there is much more information on circulating miRNA than on circulating DNA, and detection of miRNA is more sensitive and quantifiable than that of proteins, indicating miRNA may be more powerful in the diagnosing and prognosis of organ survival and immune rejection. The levels of circulating miRNAs are quite stable in healthy people, but may become deregulated once cell death occurs [15]. As mentioned above, circulating miRNAs are tissue/organ-specific and may be disease- and stage-specific, resistant to adverse conditions, easily detectable and quantifiable, and readily accessible using non-invasive methods, all of which confers on them promise for the detection of rejection.

Organ/tissue-specific/enriched miRNAs may be of more value than some ubiquitously-expressed miRNAs in organ allo- or xeno-transplantation, as they are specific indicators for the state of the transplanted organ. Circulating organ/tissue-specific/enriched miRNAs may indicate direct organ/tissue injury or cell death, and circulating immune-associated miRNAs may serve as sensors of the immune state. A combination of organ/tissue-specific/enriched miRNA and immune-associated miRNAs might be valuable to distinguish between different diseases, such as lung injury caused by cytomegalovirus (CMV) infection or immune rejection.

Therefore, this paper provides background information on circulating miRNAs as a proof of concept that circulating organ-specific miRNAs serve as non-invasive biomarkers for a wide spectrum of clinical organ-specific manifestations. We subsequently review the potential role of circulating miRNAs in organ allo (xeno)-transplantation, and finally discuss the challenges that remain if these biomarkers are to prove valuable in organ allo (xeno)-transplantation.

## 2. Background

### 2.1. Circulating miRNA: Sources and Functions

miRNA biogenesis and mechanisms of action have been reviewed in detail [25,26,27]. Other than intracellular miRNA, mature miRNA can be found in body fluids, including plasma, serum, urine, tears, breast milk, amniotic fluid, bronchial lavage, pleural fluid, cerebrospinal fluid, and saliva [15,28,29,30,31] (reviewed in [32]). Circulating miRNA may be actively secreted from living cells, but may also be passively released by dying cells. Circulating miRNA may be found in microvesicles, in apoptotic bodies, in/on high density lipoprotein (HDL) particles, or may bind to argonaute (AGO) proteins (reviewed in [32,33,34]). Mostly, miRNAs are byproducts of cellular activities and cell death, except certain miRNA species might also function in cell–cell communication (reviewed in [32]).

The major form of circulating miRNA is AGO-binding miRNA, which accounts for 90%–99% of the total circulating miRNA [35,36]. AGO proteins, as essential catalytic components of the RNA-induced silencing complex (RISC), play a central role in RNA-mediated regulation of gene expression, during which all mature miRNAs become associated with one of the four AGO proteins (mainly AGO2) [8,37]. The AGO protein provides stability to the miRNA. The function of circulating AGO-binding miRNAs is unknown.

The miRNAs in microvesicles enveloped by a phospholipid bilayer is a mixed population of exosomes and shedding vesicles (reviewed in [32]). The phospholipid bilayer is impermeable to RNases and renders stability to miRNAs [38]. Although the underlying mechanisms of sorting and secretion have not yet been fully explained, the vesicular miRNAs are suggested to be released through a ceramide-dependent secretory pathway [39,40,41]. Although accounting for only a minority of the total circulating miRNA [42,43,44], miRNAs in microvesicles play an important role in cell-to-cell or organ-to-organ communication [42,45,46], including in immune regulation (reviewed in [47,48]) and in cancer metastasis (reviewed in [49,50]). Some miRNAs are packaged into or bind to HDL particles regulated by neutral sphingomyelinase, which also transports endogenous miRNAs and delivers them to recipient cells with functional targeting capabilities [51]. MiRNAs can also be entrapped into apoptotic bodies during apoptosis and accompanied by various cellular organelles [52]. Sources of circulating miRNA are shown in Figure 1 (details shown in Table 1).

The level of circulating miRNA is quite stable in healthy people, but deregulated under certain conditions, such as physiological changes, inflammation, and cell death [15]. Hence, circulating miRNAs are promising biomarkers for specific diseases. In addition, circulating miRNAs resist harsh conditions, including RNase digestion, freeze-thawing, boiling, and extreme pH conditions, rendering them extremely promising markers for the non-invasive detection of various diseases [15,28].

### 2.2. Circulating Liver-Specific miRNAs Serve as Novel Biomarkers in Liver Disease

The liver is one of the most vital organs in humans and other animals, with a wide range of functions, including detoxification of various metabolites, protein synthesis, and the production of bile [55]. In the liver, the most abundant miRNA (>70%) is miR-122, which is also the hepatocyte-specific miRNA [12,56], acting as a novel regulator of diverse aspects of hepatic function, including metabolism [57], response to stress [58], and maintenance of hepatic phenotype [59]. Once the microenvironment changes, levels of circulating miR-122 are deregulated, for example, in certain pathological conditions such as drug- or alcohol-induced cytotoxicity, viral infections, and hepatic disease progression (reviewed in [60]).

**Figure 2:**
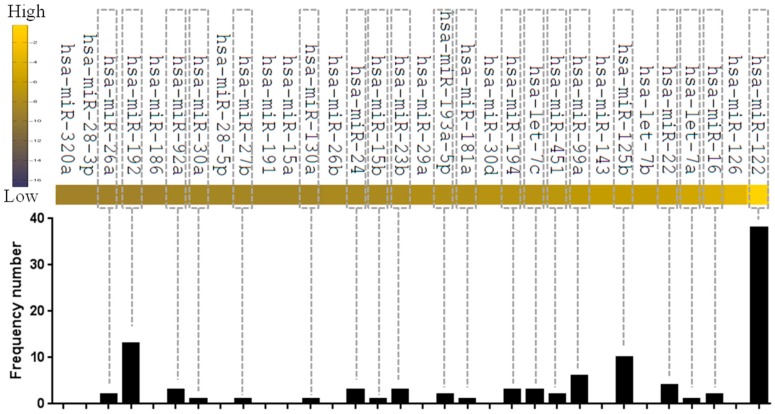
Circulating liver-specific/enriched miRNAs serve as biomarkers for different liver diseases. Expression profiles of liver miRNAs were obtained from a web-accessible database (http://www.mirz.unibas.ch/), of which miRNA expression was determined by small RNA library sequencing [13]. The frequencies of circulating miRNAs as biomarkers were determined from 65 published papers.

In recent years, a large number of reports have been published indicating that circulating miR-122 can be applied as a non-invasive biomarker for a wide spectrum of clinical liver-specific diseases, including hepatic virus infections [61,62,63,64,65,66,67,68], liver injury [69,70,71,72,73], hepatic cirrhosis [74,75], and hepatocellular carcinoma [65,76,77] (reviewed in [60]). By reviewing the published papers, we have summarized the information, and it suggests that circulating miR-122 is the most frequent biomarker for liver disease (Figure 2).

Other circulating hepatic-abundant miRNAs could also serve as biomarkers for liver disease, such as miR-22 [71,78], miR-125b [64,65,68,79], miR-99a [65,68,80] and miR-192 [72,74,76,77] (Figure 2). It is worth noting that a panel of circulating miRNAs may enhance specificity and sensitivity [76,80], in which both liver-specific miRNAs and liver-abundant miRNAs might indicate a specific type of liver disease or dysfunction.

### 2.3. Circulating Cardiac-Specific miRNAs Serve as Novel Biomarkers in Heart Disease

The heart is a muscular organ that pumps blood through the blood vessels of the circulatory system [81]. Unlike liver-specific miR-122, cardiac-specific miRNA (miR-208) is not a heart-enriched miRNA (e.g., miR-1, miR-133a, miR-499, and miR-296). Once the heart is injured, muscle-enriched miRNAs and cardiac-specific miR-208 are released into body fluids, and can serve as novel biomarkers for heart disease [82,83,84,85,86,87,88,89,90,91] (also reviewed in [92,93,94,95]). In fact, the boundary is not clear between “specific” and “enriched/abundant” miRNAs, terms which sometimes replace each other in different published papers [96].

The heart is rich in blood vessels, such as arteries, veins, and capillaries. Hence, enriched/specific miRNAs in vascular endothelial cells, such as miR-126, may also be indicators of cardiovascular diseases. For example, circulating miR-126 is significantly down-regulated in patients suffering symptomatic atherosclerosis [97], acute myocardial infarction [98], and heart failure [99].

### 2.4. Circulating Kidney-Specific miRNAs Serve as Novel Biomarkers in Renal Disease

Unlike the liver and heart, the kidney possesses more diverse cell types and structures which carry out complicated functions, including regulation of blood-electrolyte balance, maintenance of blood acid–base balance, removal of excess water-soluble wastes from the blood, and regulation of blood pressure [100]. A set of kidney-specific miRNAs is present, of which expression levels favor understanding and diagnosis for renal diseases [101]. These specific miRNAs include miR-192, miR-194, miR-204, miR-215, and miR-216, which have low expression in the liver, lung and heart. As a proof of concept, different renal cell types possess different profiles of miRNA expression [12,13], which may be indicators of different diseases in different segments of the kidney. For example, miR-192 and miR-194 are significantly more abundant in the cortex, while miR-30c and miR-200c are highly expressed in the medulla [102,103].

Surprisingly, few circulating kidney-specific miRNAs have been reported as non-invasive biomarkers for renal disease in humans, possibly because most research focused on excavating biomarkers for the more accessible sample of urine. Indeed, circulating miRNAs have rarely been studied in the diagnosis of renal diseases. We could find only one paper which reported that circulating levels of miR-16 and miR-320 are down-regulated while miR-210 is up-regulated in patients with acute kidney injury [104]. However, in rat models, plasma kidney-specific/enriched miRNAs (miR-10a, miR-192, and miR-194) have been reported to be potential biomarkers for renal ischemia-reperfusion injury [105].

Urine, which is more accessible than serum/plasma, can provide a non-invasive sample to test for renal disease. Urinary miRNAs are filtered or excreted from the kidney and/or urinary tract [28] (reviewed in [106]). Once the filtering function of the kidney is injured, miRNAs from the plasma may become urinary miRNAs, which can serve as potential biomarkers of renal disease. These miRNAs can be considered similar to certain urinary proteins which are normally absent from the urine and, when present, indicate kidney damage. Urinary miR-200a levels are useful for the diagnosis of renal tubular dysfunction (in the Dahl salt-sensitive rat with high salt administration) [107]. A high urinary level of miR-494 precedes an increase in serum creatinine in patients with kidney injury, indicating that urinary miR-494 can serve as an early and non-invasive indicator of acute renal injury [108]. Urinary miRNAs excreted from the kidney and/or urinary tract are also good sensors of disease of the urinary system. For example, a panel of miRNAs (miR-126, miR-152, miR-182) is significantly increased in the urine of patients with urothelial bladder cancer [109], which suggests that urine miRNAs may serve as markers of bladder cancer.

### 2.5. Circulating Lung-Specific miRNAs Serve as Novel Biomarkers in Lung Disease

The lung is the primary organ of the respiratory system in mammals, functioning in the process of gas exchange. By using semi-quantitative real-time RT-PCR, miR-92, miR-26a, miR-200c, miR-16, let-7b, miR-125a, and miR-125b have been found to be the most highly expressed miRNAs in human airway tissues, having levels >70-fold higher than the average miRNA and contributing 55.5% of the total mRNA detected in airway biopsies [110]. Using microarrays, miR-195 and miR-200c were found to be expressed specifically in the rat lung [111], although miR-195 is only moderately expressed in the human lung [110]. In patients suffering from idiopathic pulmonary fibrosis, miR-200c was significantly increased in sera compared to healthy controls [112]. The down-regulation of both circulating miR-195 and miR-21 predicted poor differentiation of non-small cell lung cancer [113]. We could not identify other reports, probably because relatively few studies have been carried out in this field.

## 3. The Potential Role of Circulating miRNAs to Detect Graft Rejection

### 3.1. Circulating Organ-Specific miRNAs in Organ Allotransplantation

In organ allotransplantation, the grafts may be damaged by the host immune system. Levels of certain circulating miRNAs from both the transplanted organ graft and from cells of the host’s immune system may change significantly. Indeed, there are already reports focusing on rejection of organ allografts [16,114,115,116,117,118]. Organ-specific/enriched miRNAs normally serve as biomarkers of direct injury or ischemia-reperfusion injury.

For example, hepatocyte-abundant miRNAs (miR-122, miR-148a) and cholangiocyte-abundant miRNAs (miR-30e, miR-222, and miR-296) may be elevated in the solution in which the organ has been stored and transported, which may predict the quality of a donor liver [114,115]. Serum levels of hepatocyte-derived miRNAs (miR-122, miR-148a, miR-194) were elevated in patients with liver injury and positively-correlated with aminotransferase levels in patients with liver transplants [16]. Kidney-derived miRNAs (miR-21, miR-20a, miR-146a, miR-199a-3p, miR-214, miR-192, miR-187, miR-805, and miR-194) may reveal a signature of kidney damage following ischemia-reperfusion injury, which could be used as a biomarker of renal injury [116,117]. Circulating pancreas/islet-specific miR-375 could be a reliable biomarker to detect graft damage in clinical islet transplantation, and can be compared with C-peptide and pro-insulin levels [118]. Circulating organ/tissue-specific/enriched miRNAs which can potentially be applied to monitor typical allograft rejections are shown in Table 2.

### 3.2. Circulating Immune-Associated miRNAs in Organ Allotransplantation

In contrast, circulating immune-associated miRNAs normally serve as biomarkers for immune activation and immune rejection. In patients with allotransplanted hearts, circulating miR-10a (down-regulated), miR-31, miR-92a, and miR-155 (all up-regulated) strongly discriminate between patients undergoing allograft rejection and those not undergoing rejection [120]. Notably, these miRNAs (miR-10a, miR-31, miR-92a, and miR-155) were associated with an inflammatory response [121,122,123,124]. Decreased levels of miR-210 were observed in the urine of patients undergoing acute T cell-mediated renal allograft rejection, and increased in response to corticosteroid therapy [125]. Eight miRNAs in peripheral blood mononuclear cells have been identified as sensors of operational tolerance in kidney transplant recipients (up-regulation: miR-450b-5p, miR142-3p, miR-876-3p, and miR-106b; down-regulation: miR-508-3p, miR-148b, miR-324-5p, and miR-98) [126]. In particular, up-regulation of miR-142-3p is correlated with operational tolerance of a B lymphocyte subset, which would be useful to identify immune tolerance and optimize individualized treatment by immunosuppressive drugs.

### 3.3. Circulating Organ-Specific miRNAs in Organ Xenotransplantation

Organ allotransplantation is effective in the treatment of terminal organ failure. However, >90% patients remain on the waiting list due to the shortage of deceased human donors [127]. Pigs are regarded as the most promising alternatives as sources of organs and tissues [128,129]. Progress has been made by gene-editing, which dramatically reduces immune rejection [130,131], and may reduce the potential risk of the presence of porcine endogenous retrovirus (PERV) in the pig organ [132,133,134]. However, xenotransplantation is in urgent need of novel biomarkers to monitor graft survival or immune rejection. Circulating organ-specific/enriched miRNAs may provide methods to monitor xenograft survival, and immune-associated miRNAs may serve to monitor immune rejection and immune tolerance (by non-invasive procedures).

In a pig model of acetaminophen-induced acute liver failure, pig (*Sus scrofa*)-derived miRNAs including ssc-miR-122 (liver-specific), ssc-miR-192 (kidney-specific), and ssc-miR-124-1 (brain-enriched) were associated with clinical evidence of liver, kidney and brain injury, respectively [135]. However, there are no further reports addressing this issue, probably due to limited pre-clinical experience. Lack of complete genome information further impeded profiling miRNA expression. As described from differences in miRNAs between humans and mice [13], most miRNAs are conserved in sequence and abundance between species. Indeed, there are already reports of pig miRNA sequencing [136,137,138,139,140,141,142] providing the expression profiles of different organs, tissues, and cells.

Certain miRNAs may be quite different in sequence and expressed differentially between species [136,143,144,145] (sometimes called xeno-miRNAs), which may be useful to increase specificity. For example, the pig-species miRNA ssc-miR-199b* possesses an internal 2-nt mismatch compared with its counterpart from human and mouse. It is moderately expressed in liver, heart, and lung [146], and may be discriminated by Taqman probe in qPCR. ssc-miR-199b* may potentially serve as a biomarker for the fate of a xenograft. For a xeno-miRNA to serve as a biomarker in xenotransplantation, it would be best for it to be abundant, differentiable, and easily quantifiable. Substantial work needs to be carried out to address this issue. Circulating organ/tissue-specific/enriched miRNAs that might be applied to monitor typical xenograft rejection are shown in Table 3.

## 4. Discussion—Challenges and Solutions

There remain too many gaps in our knowledge of miRNAs, including regulation of miRNA production, specific targets, and mechanisms of active secretion [148]. For example, intracellular levels of miRNAs and/or levels of secretion may fluctuate at different stages of rejection, which may complicate interpretation of the data. For example, circulating liver-specific miRNA-122 is dramatically up-regulated during liver injury [69,70,71,72,73], but significantly down-regulated during late-stage hepatic cirrhosis [75] and hepatocellular carcinoma [76,77], due to intracellular down-regulation of miRNA production. Therefore, levels of certain circulating organ-specific miRNAs may be significantly up-regulated during acute rejection, but significantly down-regulated during chronic or late rejection. Changes in circulating miRNAs cannot simply be explained by rejection increasing their levels. Substantial and critical studies need to be carried out to identify whether circulating miRNAs can be used as reliable biomarkers for diagnosis, prognosis, and response to therapy.

Another challenge we face is the problem of specificity. (1) One circulating miRNA may not be sufficiently specific to target a certain organ, tissue or cell. For example, miR-375 is pancreas/islet-specific and reported to be a reliable biomarker to detect islet graft injury [118], but we note it is also expressed in the airways [110] and thus damage to an airway may interfere with the diagnosis of islet rejection; (2) Another problem is that immune-associated miRNAs may be elevated during opportunistic infection, inflammation, and cancer, which may also confuse interpretation of the results; (3) What may provide an additional problem is that most published researchers have designed their experiments to compare a disease group with a healthy group, rather than included alternative disease groups. A biomarker may powerfully discriminate between a disease group and a healthy group but may also prove to be a biomarker for another disease. Only a subset of reported blood-based miRNA biomarkers has specificity for a particular disease [149]. Therefore, a panel of miRNAs may be more specific to determine allo- or xeno-graft survival or rejection. Some researchers applying high through-put sequencing have already shown a panel of miRNAs with enhanced specificity in diagnosis [76,80]. In addition, if it is possible to identify xeno-miRNAs in pig-to-human xenotransplantation models, this may enhance specificity.

Moreover, the sensitivity of circulating miRNAs should also be taken into consideration for different types of organ/tissue transplantation. A normal cell-turnover rate is mass-dependent [21]. Therefore, the level of a circulating miRNA may also be mass-dependent even during rejection. As reviewed above, we believe circulating organ-specific/enriched miRNAs are easily detectable during solid organ transplantations such as liver, heart, kidney, and lung. However, other transplants of small-mass tissues, such as corneal, islet, skin, and cardiac valves, may exhibit limited release of tissue-specific/enriched miRNAs during rejection. As we learnt from clinical islet allotransplantation, circulating islet-specific miR-375 is dramatically elevated during rejection [118], indicating tissue-specific/enriched miRNAs derived from small-mass tissues could be potentially detected. In addition, circulating miRNA can be found in different body fluids [15,28,29,30,31] (reviewed in [32]), thus proper choice of sampling may further enhance sensitivity.

A technical challenge is that there is currently no standardized method for measuring miRNAs. Different technologies have been applied to measure circulating miRNAs, such as high through-put sequencing, microarrays, PCR arrays, qPCR, and droplet digital PCR, which make the data difficult to compare. A high through-put, precise, and reproducible detection method is urgently needed, which may lead to increased progress in this field. In addition, the heterogeneity of circulating miRNA (in microvesicles, apoptotic bodies, HDL particles, or bound to AGO protein) requires further standardization of protocols for sample processing to make sure the data between different studies can be directly compared.

Although thousands of reports have demonstrated the potential of measuring circulating miRNA, there still is a huge gap between fundamental research and clinical application, because no standard is available by which clinicians can judge whether a graft is rejected or not. Apart from unsatisfactory specificity and sensitivity as described above, clinical variance or biases such as donor/recipient heterogeneity, immunosuppressive treatment, and potential contamination may further complicate the diagnosis. For example, the mass of the organ/tissue is variable resulting from donor heterogeneity, and levels of circulating tissue-specific/enriched miRNAs may also be variable. Therefore, it will be hard to establish a normal or healthy threshold for circulating miRNAs. As we learnt from cell-free DNA in the diagnosis of rejection [21], levels and features of circulating organ/tissue-specific/enriched miRNAs should be identified first in different recipients without evidence of graft rejection. Ideally, there should be a representative mathematical model or regression curve for recipients without graft rejection, which will then allow precise diagnosis of rejection. In this case, rejection judged by individual dynamics of circulating miRNAs may be more applicable than the fold-change normally reported in most fundamental studies. This issue needs further study.

Nevertheless, circulating organ-specific miRNAs have the potential to provide biomarkers for various diseases and as non-invasive indicators of organ/tissue allograft and/or xenograft rejection.

## Figures and Tables

**Table 1:** Types, sources, functions, content, and size of different types of circulating miRNA.

Types	Sources	Functions	Content	Size	Citation
AGO-binding	mainly necrotic cells ^a^	byproducts	90%–99%	unknown	[35,36]
exosomes	living cells	cell-to-cell communication	minority	30–100 nm	[39,40,41,42,43,44,45,46]
shedding vesicles	living cells	cell-to-cell communication	minority	0.1–1 μm	[32,53,54]
HDL particles	living cells	cell-to-cell communication	minority	8–12 nm	[51]
apoptotic bodies	apoptotic cells	byproducts	minority ^b^	1–4 μm	[52]

^a^ Circulating AGO-binding miRNAs may also be actively secreted from living cells; ^b^ The content may increase to some extent under disease conditions.

**Table 2:** Organ/tissue-specific/enriched miRNAs in organs/tissue of humans (*Homo sapiens*).

Organ/Tissue	Specific/Enriched miRNAs ^a^	Citation
Liver	**miR-122**, miR-125b, miR-16, miR-99a	[12,56]
Heart	miR-1, miR-126, miR-133a, **miR-208**, miR-296, miR-499	[82,83,84,85,86,87,88,89,90,91]
Kidney	**miR-192**, **miR-194**, **miR-204**, **miR-215**, **miR-216**	[101]
Lung	let-7b, miR-125a, miR-125b, miR-16, **miR-195**, **miR-200c**, miR-26a, miR-92	[110,111]
Pancreas/Islet	**miR-375**	[13,119]

^a^ All the organ/tissue-specific miRNAs are underlined and in bold.

**Table 3:** Organ/tissue-specific/enriched miRNAs in typical organs/tissue of pigs (*Sus scrofa*).

Organ/Tissue	Specific/Enriched miRNAs ^a^	Xeno-miRNAs	Citation
Liver	**miR-122**, miR-153-3p, miR-194	miR-199b*	[136,137,146]
Heart	miR-1, miR-133, **miR-208**, **miR-499**	miR-199b*	[136,146]
Kidney	miR-125b, **miR-192**, miR-200a, miR-23b	unknown	[139,147]
Lung	let-7i, miR-143-3p, miR-145, miR-320	miR-199b*	[138,139,146]

^a^ All the organ/tissue-specific miRNAs are underlined and in bold.

## Abbreviations

AGO	argonaute protein
HDL	high density lipoprotein
hsa	*Homo sapiens*
miRNA(miR-)	MicroRNA
PCR	polymerase chain reaction
qPCR	quantitative PCR
RNA	ribonucleic acid
RISC	RNA-induced silencing complex
ssc	*Sus scrofa*
CMV	Cytomegalovirus
